# Supplementary material for: Meta-Analysis and Gene Set Enrichment Relative to ER Status Reveal Elevated Activity of MYC and E2F in the “Basal” Breast Cancer Subgroup
Source: PLoS One. 2009 Mar 9;4(3):e4710. doi: 10.1371/journal.pone.0004710 (PMC2650420; doi:10.1371/journal.pone.0004710)
Supplement: Dataset S1 — GSEA results from first screen. The file contains a zipped archive of the HTML results of the second GSEA screen. Reports for enriched or depleted datasets may be accessed from the “index.html” link within the my_analysis.GseaPreranked.1219975950625.rpt.zip ZIP archive. Enrichment plots are available for each of the 50 most enriched and depleted gene sets. (13.32 MB ZIP) [file pone.0004710.s001.zip › my_analysis.GseaPreranked.1219975950625.rpt.zip!//CHR3P21.html]

Details for gene set CHR3P21[GSEA]

|  || Dataset | zSco.ERneg.to.ERpos\_collapsed |
| Phenotype | NoPhenotypeAvailable |
| Upregulated in class | na\_neg |
| GeneSet | CHR3P21 |
| Enrichment Score (ES) | -0.48700038 |
| Normalized Enrichment Score (NES) | -2.412034 |
| Nominal p-value | 0.0 |
| FDR q-value | 0.0 |
| FWER p-Value | 0.0 |
Table: GSEA Results Summary

  

Fig 1: Enrichment plot: CHR3P21      
 Profile of the Running ES Score & Positions of GeneSet Members on the Rank Ordered List

  

| PROBE | GENE SYMBOL | GENE\_TITLE | RANK IN GENE LIST | RANK METRIC SCORE | RUNNING ES | CORE ENRICHMENT || 1 | ITGA9 |  |  | 258 | 5.072 | -0.0052 | No |
| 2 | CDC25A |  |  | 281 | 4.993 | 0.0073 | No |
| 3 | TMEM158 |  |  | 290 | 4.964 | 0.0208 | No |
| 4 | NT5DC2 |  |  | 453 | 4.499 | 0.0212 | No |
| 5 | DUSP7 |  |  | 597 | 4.159 | 0.0222 | No |
| 6 | CCR1 |  |  | 689 | 3.952 | 0.0265 | No |
| 7 | CXCR6 |  |  | 1879 | 2.624 | -0.0565 | No |
| 8 | CSPG5 |  |  | 1934 | 2.583 | -0.0533 | No |
| 9 | HYAL1 |  |  | 2022 | 2.511 | -0.0527 | No |
| 10 | RNU3IP2 |  |  | 2065 | 2.473 | -0.0489 | No |
| 11 | RAD54L2 |  |  | 2093 | 2.457 | -0.0440 | No |
| 12 | DOCK3 |  |  | 2195 | 2.390 | -0.0449 | No |
| 13 | ENDOGL1 |  |  | 2416 | 2.245 | -0.0552 | No |
| 14 | TMF1 |  |  | 2481 | 2.211 | -0.0538 | No |
| 15 | SCN5A |  |  | 2532 | 2.183 | -0.0514 | No |
| 16 | MAP4 |  |  | 2611 | 2.140 | -0.0513 | No |
| 17 | DLEC1 |  |  | 2613 | 2.138 | -0.0453 | No |
| 18 | NCKIPSD |  |  | 2631 | 2.129 | -0.0405 | No |
| 19 | PCBP4 |  |  | 3006 | 1.928 | -0.0635 | No |
| 20 | TRAIP |  |  | 3007 | 1.928 | -0.0580 | No |
| 21 | ABHD6 |  |  | 3029 | 1.919 | -0.0542 | No |
| 22 | PTK9L |  |  | 3060 | 1.903 | -0.0511 | No |
| 23 | CDCP1 |  |  | 3187 | 1.847 | -0.0554 | No |
| 24 | STAB1 |  |  | 3300 | 1.793 | -0.0588 | No |
| 25 | GPR27 |  |  | 3306 | 1.791 | -0.0541 | No |
| 26 | CCDC71 |  |  | 4044 | 1.496 | -0.1059 | No |
| 27 | GRM2 |  |  | 4398 | 1.355 | -0.1289 | No |
| 28 | ID2B |  |  | 4442 | 1.338 | -0.1283 | No |
| 29 | CAMKV |  |  | 4647 | 1.270 | -0.1403 | No |
| 30 | GNL3 |  |  | 4741 | 1.239 | -0.1438 | No |
| 31 | SLC22A13 |  |  | 4772 | 1.230 | -0.1426 | No |
| 32 | CCR3 |  |  | 5297 | 1.049 | -0.1795 | No |
| 33 | PPP2R5C |  |  | 5301 | 1.047 | -0.1767 | No |
| 34 | SEMA3B |  |  | 5336 | 1.032 | -0.1764 | No |
| 35 | CCR2 |  |  | 5356 | 1.025 | -0.1749 | No |
| 36 | SLC38A3 |  |  | 5522 | 0.967 | -0.1847 | No |
| 37 | SLC22A14 |  |  | 5563 | 0.954 | -0.1850 | No |
| 38 | ALS2CL |  |  | 5659 | 0.927 | -0.1896 | No |
| 39 | CCR9 |  |  | 5780 | 0.885 | -0.1962 | No |
| 40 | GNAT1 |  |  | 5961 | 0.830 | -0.2076 | No |
| 41 | PB1 |  |  | 5977 | 0.822 | -0.2064 | No |
| 42 | SCN11A |  |  | 6036 | 0.798 | -0.2085 | No |
| 43 | HYAL3 |  |  | 6351 | 0.701 | -0.2304 | No |
| 44 | KLHL18 |  |  | 6369 | 0.695 | -0.2297 | No |
| 45 | SCN10A |  |  | 6389 | 0.689 | -0.2292 | No |
| 46 | IFRD2 |  |  | 6869 | 0.528 | -0.2641 | No |
| 47 | LRRC2 |  |  | 6874 | 0.526 | -0.2629 | No |
| 48 | ITIH1 |  |  | 6968 | 0.499 | -0.2686 | No |
| 49 | MOBP |  |  | 7165 | 0.430 | -0.2823 | No |
| 50 | TNNC1 |  |  | 7187 | 0.422 | -0.2827 | No |
| 51 | CTNNB1 |  |  | 7241 | 0.405 | -0.2855 | No |
| 52 | TREX1 |  |  | 7354 | 0.365 | -0.2930 | No |
| 53 | SETD2 |  |  | 7379 | 0.353 | -0.2938 | No |
| 54 | PHF7 |  |  | 7460 | 0.325 | -0.2990 | No |
| 55 | ALAS1 |  |  | 7540 | 0.292 | -0.3042 | No |
| 56 | TMEM113 |  |  | 7574 | 0.280 | -0.3059 | No |
| 57 | SLC6A20 |  |  | 7601 | 0.269 | -0.3071 | No |
| 58 | FEZF2 |  |  | 7700 | 0.231 | -0.3139 | No |
| 59 | SS18L2 |  |  | 7916 | 0.140 | -0.3298 | No |
| 60 | DNASE1L3 |  |  | 8153 | 0.055 | -0.3476 | No |
| 61 | CCBP2 |  |  | 8184 | 0.044 | -0.3498 | No |
| 62 | ZNF197 |  |  | 8197 | 0.037 | -0.3506 | No |
| 63 | ZNF35 |  |  | 8235 | 0.020 | -0.3533 | No |
| 64 | ZNF589 |  |  | 8305 | -0.000 | -0.3586 | No |
| 65 | PRKAR2A |  |  | 8390 | -0.033 | -0.3649 | No |
| 66 | WDR48 |  |  | 8413 | -0.042 | -0.3664 | No |
| 67 | COL7A1 |  |  | 8528 | -0.092 | -0.3748 | No |
| 68 | CTDSPL |  |  | 8706 | -0.173 | -0.3878 | No |
| 69 | VPRBP |  |  | 8809 | -0.212 | -0.3950 | No |
| 70 | XCR1 |  |  | 8816 | -0.216 | -0.3948 | No |
| 71 | ARIH2 |  |  | 8923 | -0.273 | -0.4021 | No |
| 72 | ITIH3 |  |  | 8976 | -0.304 | -0.4052 | No |
| 73 | ARMET |  |  | 9047 | -0.334 | -0.4095 | No |
| 74 | WNT5A |  |  | 9278 | -0.447 | -0.4258 | No |
| 75 | LIMD1 |  |  | 9308 | -0.458 | -0.4267 | No |
| 76 | IL17RB |  |  | 9441 | -0.535 | -0.4352 | No |
| 77 | FBXW12 |  |  | 9584 | -0.608 | -0.4443 | No |
| 78 | CACNA2D3 |  |  | 9627 | -0.632 | -0.4457 | No |
| 79 | TMEM103 |  |  | 9733 | -0.686 | -0.4517 | No |
| 80 | HESX1 |  |  | 9790 | -0.718 | -0.4539 | No |
| 81 | TUSC4 |  |  | 9822 | -0.739 | -0.4542 | No |
| 82 | BSN |  |  | 9932 | -0.798 | -0.4602 | No |
| 83 | ZNF167 |  |  | 10130 | -0.919 | -0.4726 | No |
| 84 | MAPKAPK3 |  |  | 10168 | -0.939 | -0.4727 | No |
| 85 | ZDHHC3 |  |  | 10216 | -0.966 | -0.4735 | No |
| 86 | EXOSC7 |  |  | 10276 | -1.007 | -0.4752 | No |
| 87 | RPL29 |  |  | 10303 | -1.032 | -0.4742 | No |
| 88 | LARS2 |  |  | 10354 | -1.064 | -0.4750 | No |
| 89 | GLB1 |  |  | 10363 | -1.070 | -0.4726 | No |
| 90 | CADPS |  |  | 10554 | -1.210 | -0.4836 | Yes |
| 91 | SCAP |  |  | 10557 | -1.213 | -0.4803 | Yes |
| 92 | HYAL2 |  |  | 10569 | -1.220 | -0.4776 | Yes |
| 93 | ATXN7 |  |  | 10573 | -1.222 | -0.4744 | Yes |
| 94 | MST1R |  |  | 10574 | -1.223 | -0.4709 | Yes |
| 95 | ITIH4 |  |  | 10731 | -1.335 | -0.4790 | Yes |
| 96 | DAG1 |  |  | 10780 | -1.375 | -0.4788 | Yes |
| 97 | PTPRG |  |  | 10812 | -1.396 | -0.4771 | Yes |
| 98 | GPX1 |  |  | 10856 | -1.433 | -0.4763 | Yes |
| 99 | SLC26A6 |  |  | 10914 | -1.489 | -0.4765 | Yes |
| 100 | CELSR3 |  |  | 10927 | -1.504 | -0.4731 | Yes |
| 101 | ARF4 |  |  | 11030 | -1.595 | -0.4763 | Yes |
| 102 | IHPK2 |  |  | 11047 | -1.612 | -0.4730 | Yes |
| 103 | IHPK1 |  |  | 11178 | -1.729 | -0.4779 | Yes |
| 104 | RPP14 |  |  | 11189 | -1.736 | -0.4738 | Yes |
| 105 | RBM15B |  |  | 11260 | -1.794 | -0.4740 | Yes |
| 106 | CAMP |  |  | 11384 | -1.917 | -0.4779 | Yes |
| 107 | USP19 |  |  | 11423 | -1.959 | -0.4752 | Yes |
| 108 | PLXNB1 |  |  | 11444 | -1.986 | -0.4711 | Yes |
| 109 | NAT6 |  |  | 11447 | -1.987 | -0.4656 | Yes |
| 110 | CX3CR1 |  |  | 11487 | -2.019 | -0.4629 | Yes |
| 111 | GNAI2 |  |  | 11509 | -2.046 | -0.4587 | Yes |
| 112 | CCDC72 |  |  | 11557 | -2.092 | -0.4563 | Yes |
| 113 | WDR6 |  |  | 11563 | -2.098 | -0.4507 | Yes |
| 114 | ACTR8 |  |  | 11601 | -2.126 | -0.4475 | Yes |
| 115 | UBE1L |  |  | 11613 | -2.136 | -0.4423 | Yes |
| 116 | ENTPD3 |  |  | 11688 | -2.209 | -0.4416 | Yes |
| 117 | AMT |  |  | 11718 | -2.239 | -0.4375 | Yes |
| 118 | TMEM110 |  |  | 11743 | -2.260 | -0.4329 | Yes |
| 119 | SFMBT1 |  |  | 11807 | -2.337 | -0.4310 | Yes |
| 120 | MST1 |  |  | 11836 | -2.356 | -0.4265 | Yes |
| 121 | SACM1L |  |  | 11921 | -2.459 | -0.4259 | Yes |
| 122 | RASSF1 |  |  | 12004 | -2.564 | -0.4248 | Yes |
| 123 | NBEAL2 |  |  | 12048 | -2.634 | -0.4206 | Yes |
| 124 | BAP1 |  |  | 12070 | -2.658 | -0.4147 | Yes |
| 125 | RBM6 |  |  | 12077 | -2.668 | -0.4076 | Yes |
| 126 | CCDC51 |  |  | 12106 | -2.711 | -0.4020 | Yes |
| 127 | UQCRC1 |  |  | 12149 | -2.773 | -0.3973 | Yes |
| 128 | RBM5 |  |  | 12151 | -2.775 | -0.3895 | Yes |
| 129 | SEMA3G |  |  | 12221 | -2.872 | -0.3866 | Yes |
| 130 | C3ORF18 |  |  | 12316 | -2.986 | -0.3853 | Yes |
| 131 | VILL |  |  | 12407 | -3.111 | -0.3833 | Yes |
| 132 | ACY1 |  |  | 12449 | -3.179 | -0.3774 | Yes |
| 133 | NEK4 |  |  | 12490 | -3.249 | -0.3712 | Yes |
| 134 | CACNA2D2 |  |  | 12518 | -3.292 | -0.3639 | Yes |
| 135 | ARHGEF3 |  |  | 12607 | -3.445 | -0.3608 | Yes |
| 136 | MYRIP |  |  | 12635 | -3.502 | -0.3529 | Yes |
| 137 | GLT8D1 |  |  | 12663 | -3.549 | -0.3449 | Yes |
| 138 | NISCH |  |  | 12684 | -3.584 | -0.3362 | Yes |
| 139 | USP4 |  |  | 12702 | -3.625 | -0.3272 | Yes |
| 140 | QARS |  |  | 12704 | -3.626 | -0.3170 | Yes |
| 141 | FYCO1 |  |  | 12720 | -3.654 | -0.3078 | Yes |
| 142 | TUSC2 |  |  | 12728 | -3.684 | -0.2979 | Yes |
| 143 | NME6 |  |  | 12789 | -3.854 | -0.2915 | Yes |
| 144 | RHOA |  |  | 12790 | -3.855 | -0.2805 | Yes |
| 145 | HEMK1 |  |  | 12808 | -3.909 | -0.2707 | Yes |
| 146 | DCP1A |  |  | 12832 | -3.962 | -0.2612 | Yes |
| 147 | SEMA3F |  |  | 12861 | -4.029 | -0.2519 | Yes |
| 148 | QRICH1 |  |  | 12882 | -4.095 | -0.2418 | Yes |
| 149 | TEX264 |  |  | 12885 | -4.101 | -0.2303 | Yes |
| 150 | ZMYND10 |  |  | 12904 | -4.161 | -0.2199 | Yes |
| 151 | PDHB |  |  | 12922 | -4.217 | -0.2092 | Yes |
| 152 | LZTFL1 |  |  | 12950 | -4.317 | -0.1990 | Yes |
| 153 | LAMB2 |  |  | 12959 | -4.349 | -0.1873 | Yes |
| 154 | SLC25A20 |  |  | 12966 | -4.379 | -0.1753 | Yes |
| 155 | TCTA |  |  | 12972 | -4.387 | -0.1632 | Yes |
| 156 | APEH |  |  | 13003 | -4.464 | -0.1528 | Yes |
| 157 | APPL |  |  | 13059 | -4.678 | -0.1437 | Yes |
| 158 | GMPPB |  |  | 13078 | -4.747 | -0.1316 | Yes |
| 159 | C3ORF60 |  |  | 13103 | -4.833 | -0.1197 | Yes |
| 160 | IMPDH2 |  |  | 13108 | -4.860 | -0.1062 | Yes |
| 161 | SPCS1 |  |  | 13178 | -5.233 | -0.0966 | Yes |
| 162 | PH-4 |  |  | 13179 | -5.236 | -0.0818 | Yes |
| 163 | CYB561D2 |  |  | 13255 | -6.022 | -0.0704 | Yes |
| 164 | CISH |  |  | 13271 | -6.217 | -0.0539 | Yes |
| 165 | TMEM115 |  |  | 13286 | -6.372 | -0.0368 | Yes |
| 166 | ABHD14A |  |  | 13303 | -6.718 | -0.0190 | Yes |
| 167 | DALRD3 |  |  | 13312 | -7.117 | 0.0006 | Yes |
Table: GSEA details [plain text format]

  

Fig 2: CHR3P21: Random ES distribution      
 Gene set null distribution of ES for **CHR3P21**
